# Ability Emotional Intelligence and Subjective Happiness in Adolescents: The Role of Positive and Negative Affect

**DOI:** 10.3390/jintelligence11080166

**Published:** 2023-08-16

**Authors:** Desirée Llamas-Díaz, Rosario Cabello, Raquel Gómez-Leal, María José Gutiérrez-Cobo, Alberto Megías-Robles, Pablo Fernández-Berrocal

**Affiliations:** 1 Department of Basic Psychology, Faculty of Psychology, Campus de Teatinos, University of Málaga, 29071 Málaga, Spain; dlld@uma.es (D.L.-D.); raqgomlea@uma.es (R.G.-L.); amegias@uma.es (A.M.-R.); berrocal@uma.es (P.F.-B.); 2 Department of Developmental and Educational Psychology, Faculty of Psychology, Campus de Teatinos, University of Málaga, 29071 Málaga, Spain; mjgc@uma.es

**Keywords:** emotional intelligence, positive affect, negative affect, subjective happiness, TIEFBA, PANAS, adolescence, ability EI, gender

## Abstract

Adolescence is an increasingly vulnerable period for the onset of affective disorders and other mental health issues that can significantly impact an individual’s subjective well-being. This study aims to examine the relationship between emotional intelligence (ability EI), measured with a performance-based instrument, and Subjective Happiness in adolescents. It also explores the mediating role of positive (PA) and negative affect (NA) in this association and the moderating role of gender. The sample consisted of 333 first-year secondary school students from five centers in Spain, with an average age of 12.11 years (SD = 0.64), ranging from 11–14 years. Path analysis revealed an indirect effect (through NA and PA jointly) of Total Ability EI on Subjective Happiness and a positive direct effect that was observed only in females. Furthermore, this association was explored through various branches of ability EI. The results of this study suggest that interventions aimed at improving emotional abilities in adolescents while modulating the intensity of their emotions could significantly impact their overall well-being.

## 1. Introduction

According to UNICEF (2021), half of mental health disorders begin in middle adolescence, at around 14 years of age. In particular, in Spain, 15% of adolescents show “severe or moderately severe” symptoms of depression and 10.8% report suicidal ideation. Therefore, calls have been made for reinforcing the role of educational centers concerning issues related to affectivity, emotions, and mental health care (UNICEF 2022). This notion makes sense since emotional intervention appears to be a protective variable for well-being in adolescents (Castillo-Gualda et al. 2018; Durlak et al. 2022; Taylor et al. 2017). A recent meta-analysis of 41 articles has linked emotional intelligence (EI)—a set of abilities related to one’s and others’ emotions—with greater subjective well-being in adolescence (Llamas-Díaz et al. 2022). Moreover, a meta-analysis assessing the effectiveness of school-based social and emotional learning (SEL) programs for students from early childhood through to high school found that the mean effects were consistently significant across various outcomes. These outcomes included improvements in SEL skills, attitudes, prosocial behaviors, and academic achievement and reductions in behavioral problems and emotional distress (Durlak et al. 2022). Furthermore, the impact of emotional learning on adolescents has been demonstrated not only in academic contexts but also in clinical settings (Daros et al. 2021). Considering the indicated needs, our general objective is to explore the relationship between ability EI and subjective well-being in adolescent females and males.

### 1.1. Emotional Intelligence

EI was defined in 1997 as “the ability to perceive accurately, appraise, and express emotion; the ability to access and/or generate feelings when they facilitate thought; the ability to understand emotion and emotional knowledge; and the ability to regulate emotions to promote emotional and intellectual growth” (Mayer et al. 1997, p. 10). Since then, EI has grown in popularity, and many definitions and assessment methods have emerged. In this regard, trait and ability EI were the first categories created in this field (Petrides and Furnham 2000; Siegling et al. 2015). However, Joseph and Newman (2010) presented a more detailed distinction between three EI models: The self-report mixed model, the self-report ability model, and the performance-based ability model. These models are characterized according to how EI is conceptualized (ability vs. mixed) and the types of instruments used (self-report vs. performance-based). Given that prior studies have found weak convergent validity between the models (Brackett et al. 2006; Joseph and Newman 2010; Webb et al. 2013) and that the literature has produced inconsistent results regarding behavioral predictions (Gómez-Leal et al. 2018; Gong and Jiao 2019; Gutiérrez-Cobo et al. 2017; O’Boyle et al. 2011), it is crucial to take into account these variations when interpreting EI.

The self-report mixed model adopts a broader conceptualization of EI that includes motivations, mental abilities, interpersonal and intrapersonal skills, and personality traits (Mayer et al. 2008). This model makes use of instruments with subscales for various personality, social, and personal well-being traits (among many others). The *Bar-On Emotional Quotient Inventory* (EQ-I; Bar-On 1997) or the *Trait Emotional Intelligence Questionnaire* (TEIQue; Petrides et al. 2006) are representative tests of the self-report mixed model for both adults and adolescents. 

The self-report ability model and the performance-based ability model understand EI as a set of emotion-related abilities, specifically those defined by Mayer and Salovey (1997). However, these two models differ in the instruments they use:

On the one hand, the self-report ability uses self-report instruments with no correct or incorrect answers (therefore, this model focuses on participants’ subjective perceptions). The *Trait Meta-Mood Scale* (TMMS; Salovey et al. 1995) is a typical test of this EI model used in adults and adolescents (Salguero et al. 2010). The relationship between gender and EI remains unclear when using self-reports. While some studies have indicated a positive correlation, others have reported a negative or non-existent relationship (Brackett et al. 2006; Cabello et al. 2016; Fernández-Berrocal et al. 2018).

On the other hand, the performance-based ability model applies instruments where participants must solve problems with correct and incorrect answers (Mayer et al. 2000). The most widely used performance test of this ability model is *the Mayer-Salovey-Caruso Emotional Intelligence Test* (MSCEIT; Mayer et al. 2002). However, this has routinely been used in adults as opposed to adolescent samples, in spite of there being a version designed for the latter (MSCEIT-YRV; Mayer et al. 2002). Given the need for more ability tests of this model, other performance tests have been created and validated for the adolescent population, such as the *Botín Foundation’s Emotional Intelligence Test for Adolescents* (TIEFBA; Fernández-Berrocal et al. 2011). The TIEFBA evaluates the actual performance level of EI abilities that each teenager possesses. This feature distinguishes the instrument from other measures of EI based on self-perceptions, which helps eliminate the biases that often affect self-report measures, such as response style or social desirability (Fernández-Berrocal 2015). In this article, our primary focus will be on performance-based ability, utilizing the TIEFBA. We have chosen this approach due to its advantages over self-report methods and the growing need in the adolescent literature for data obtained through performance-based assessments. Regarding gender differences, research suggests that females have higher total ability EI when measured using performance-based instruments (Extremera et al. 2006). However, when examining specific branches of EI, mixed results have been found. While some studies indicate that adult females outperform males in all dimensions of performance-based EI (Mayer et al. 2002; Palmer et al. 2005), another study found that females excel in the branches of facilitating, understanding, and managing, but not in perceiving (Extremera et al. 2006). In the case of adolescents, inconclusive results have also been found. One study reported gender differences in performance-based EI in favor of females in all branches and areas (Fernández-Berrocal et al. 2018). However, another study found differences only in understanding and managing emotions (Zeidner et al. 2016). Despite these investigations, relatively few studies have extensively analyzed the role of gender in different branches of EI. As a result, the influence of gender on EI—particularly in adolescents—remains unclear.

### 1.2. Subjective Well-Being: Subjective Happiness and Affect

The most widely studied dimensions of well-being include affect and happiness (Diener et al. 2018; Lyubomirsky et al. 2005). Affect indicates people’s positive and negative emotions (Diener et al. 1999) and is composed of Positive Affect (PA) or pleasant emotions, and Negative Affect (NA) or unpleasant emotions. The *Positive and Negative Affect Schedule* (PANAS; Watson et al. 1988) is one of the most widely used tests to evaluate these components. Individuals who experience PA generally feel content, connected, energized, confident, enthusiastic, and self-assured. In addition, they exhibit traits such as optimism, extroversion, and resilience. Conversely, individuals who experience NA may feel sadness, apathy, disinterest, shame, envy, and guilt. Such individuals may also have difficulty coping with stress, experience changes in physiological functioning, and have difficulties when faced with challenging environments (Crawford and Henry 2004). Meanwhile, happiness can be defined as a life with more pleasant than unpleasant experiences and a strong sense of life satisfaction (Schimmack et al. 2004) rather than an isolated pleasant feeling. For this study, we adopted a comprehensive definition of happiness that encompasses both emotional and cognitive aspects, as outlined in Lyubomirsky’s approach (Lyubomirsky et al. 2005; Lyubomirsky and Lepper 1999) and we used the *Subjective Happiness Scale* (SHS; Lyubomirsky and Lepper 1999), which is a representative test of this conceptualization.

The literature reveals that PA, NA, and subjective happiness have been treated independently (Busseri and Sadava 2011; Cabello and Fernandez-Berrocal 2015; Diener et al. 2018). One of the reasons for this approach is that subjective happiness refers to a more global measure of subjective well-being, while affect has generally been interpreted as the intensity of a person’s positive or negative emotions at a given moment (e.g., PANAS). Moreover, relationships between the components of affect and subjective happiness have been established. For instance, greater subjective happiness (assessed as an overall perception of subjective well-being) has been related to higher PA and lower NA (Bhutoria and Hooja 2018; Cheng and Furnham 2003; Gutiérrez-Cobo et al. 2021; Lyubomirsky et al. 2005; Singh and Jha 2008). Furthermore, existing experimental and cross-cultural research has provided support for the causal impact of affectivity on subjective well-being assessments (Kuppens et al. 2008; Schwarz and Clore 2007).

Concerning gender differences in the well-being of adolescents, some studies have shown females to have lower subjective well-being than males (Goldbeck et al. 2007; Moksnes and Espnes 2013), while others have reported no differences (e.g., Casas et al. 2007). However, these differences begin to emerge significantly at the age of 13–15 (Esteban-Gonzalo et al. 2020), confirming that subjective well-being might decrease as respondents reach late adolescence (Chui and Wong 2016). Nevertheless, more research is needed to clarify the relationship between gender and well-being.

### 1.3. Relationship between EI and Well-Being in Adolescents

An extensive body of evidence suggests that adolescents with higher EI present lower levels of depression and anxiety (Fernández-Berrocal et al. 2006; Gómez-Baya et al. 2017) and better psychosocial adjustment (Sanchez-Ruiz and Baaklini 2018; Inglés et al. 2014; Resurrección et al. 2014; Vega et al. 2022). Moreover, according to a systematic review examining EI and suicidal behaviors at various ages, a greater level of EI appears to play a substantial role in preventing suicidal behavior (Domínguez-García and Fernández-Berrocal 2018). Previous studies have also specifically examined the relationship between EI and subjective well-being. In adults, Sánchez-Álvarez et al. (2016) found a positive association between EI and subjective well-being, including happiness, life satisfaction, and positive affect. Furthermore, MacCann et al. (2020) studied the relationship between affect and the four branches of EI within the performance-based model. Their findings demonstrated that all four branches of EI were linked to decreased NA, while only emotion management was connected to increased PA in adolescents. In addition, higher EI is generally linked to greater subjective well-being across all dimensions and conceptualizations (Llamas-Díaz et al. 2022). Furthermore, adolescents with higher EI report feeling happier (Abdollahi et al. 2015; Platsidou 2013; Tejada-Gallardo et al. 2022) and experience more PA and less NA (Gómez-Baya and Mendoza 2018; Zhao et al. 2020). These findings have been confirmed when EI is evaluated both in mixed and self-report ability models (Koydemir and Schütz 2012; Llamas-Díaz et al. 2022; Salovey and Mayer 1990; Sánchez-Álvarez et al. 2015). However, relatively few studies have utilized performance-based instruments to confirm these relationships within this model. In fact, according to the meta-analysis by Llamas-Díaz et al. 2022, only two cross-sectional studies with adolescents used the MSCEIT. Both studies had relatively small sample sizes (N = 164 and 205, respectively), and their data revealed no significant relationship between EI and adolescent well-being. It is also relevant to note that these studies applied the adult version of the MSCEIT to their adolescent samples. Therefore, the relationship between EI measured with performance instruments and adolescent subjective well-being is unclear. 

Concerning the mechanisms underlying the relationship between EI and components of subjective well-being, the literature has confirmed the mediating role of affectivity in the relationship between self-reported EI and subjective well-being (life satisfaction) in both undergraduate (Extremera and Rey 2016; Kong and Zhao 2013) and adolescent samples (Sánchez-Álvarez et al. 2015). It is important to highlight that life satisfaction and happiness are both measures of well-being that involve a cognitive assessment of how satisfied individuals are with their lives. These constructs are strongly correlated and often overlap in the literature (Diener et al. 2018). 

Considering that EI is the mental capacity to process, analyze, and understand emotional information, when individuals enhance their emotional abilities, they can alter the overall balance of their emotional experiences (e.g., more pleasant than unpleasant), resulting in a more positive perception of their lives (Extremera and Rey 2016; Zeidner et al. 2012). Furthermore, several other studies have shown that affect mediates the relationship between EI and variables such as mind-wandering, aggression, or academic performance (Gutiérrez-Cobo et al. 2023a; Gutiérrez-Cobo et al. 2018; Martínez-Monteagudo et al. 2019; Megías et al. 2018; Salavera and Usán 2020). However, it is important to note that much of this information regarding the mediating role of affect is primarily based on self-report instruments, which do not directly assess individuals’ emotional skills. Consequently, there is a possibility of introducing unrelated factors into the evaluation of emotional processing (Brackett et al. 2006). 

Studying the relationship between EI and subjective happiness in adolescents is particularly important due to the ongoing cognitive and emotional development that takes place during this period. Adolescence presents unique challenges since not all emotional skills have yet been acquired, so it is crucial to identify which emotional skills contribute most significantly to happiness through changes in affect. Additionally, understanding the role of gender in this relationship is essential for gaining valuable insights into potential interventions aimed at improving the personal well-being of adolescents. The role of gender as a moderator between performance-based EI and subjective happiness has not been extensively studied, and the results remain unclear for both adults (Salguero et al. 2012) and adolescents (Llamas-Díaz et al. 2022). To our knowledge, this is the first study to explore the role of gender as a moderator between performance-based EI and subjective happiness in adolescents.

### 1.4. Objectives and Hypothesis 

The relationship between subjective well-being and EI in adolescents is inconclusive when ability-performance tests are used since most of the results obtained from previous studies are based on samples that use self-report measures. Moreover, the objectives of this study are also prompted by the high rate of emotional disorders in adolescents and the potential for developing intervention strategies based on the relationship between ability EI and personal well-being in this group. The primary aim of this research was to examine the relationship between ability-based EI and subjective happiness, while also investigating the mediating role of PA and NA in this relationship. To provide a comprehensive analysis of this issue, we investigated the relationship between each of the TIEFBA branches (EI abilities) and Subjective Happiness. Moreover, given the gender disparities revealed by previous studies regarding our variables of interest, we explored the moderating role of gender in these relationships. We expected to find:

1. A positive relationship between Total Ability EI and Subjective Happiness.
2. A positive relationship between Total Ability EI and PA, and a negative relationship between Total Ability EI and NA.
3. A positive relationship between Subjective Happiness and PA, and a negative relationship between Subjective Happiness and NA.
4. Total Ability EI will have a positive indirect effect on Subjective Happiness via PA and NA and significant relationships will depend on the TIEFBA branches.
5. Gender will have a moderating effect on the relationship between the variables examined.

## 2. Method

### 2.1. Participants

The sample was recruited from a non-clinical, general population, and consisted of 333 first-year students from five Spanish secondary schools located in Santander and Madrid. Of the total sample, 171 (51.4%) were males and 162 (48.6%) were females. The mean age of the overall sample was 12.11 (SD = 0.64) with a range of 11–14 years. For males, the mean age was 12.15 years (SD = 0.64), and for females, it was 12.07 years (SD = 0.61). 

### 2.2. Procedure

Parents gave their consent for the adolescents to participate. Parents and adolescents were informed about protecting the data collected (perseveration of confidentiality and anonymity), and all participants were treated following the Helsinki Declaration (Williams 2008). The Research Ethics Committee of the University of Málaga approved this research as part of the project “*Factores protectores del bienestar personal y escolar en la adolescencia. UMA18-FEDERJA-114*” (Approval Number: CEUMA: 38-2020-H). 

Participants were asked to complete questionnaires that evaluated ability EI, PA, NA, and Subjective Happiness through the online platform Lime survey (http://limesurvey.org, accessed on 8 August 2023). Online questionnaires were completed during school hours in their classroom in a single session lasting 45 min. During the evaluation, adolescents were supervised by one researcher and their teacher. Throughout the session, the researcher was always available to answer questions and support participants with reading difficulties.

### 2.3. Instruments 

*Botín Foundation’s Emotional Intelligence Test for Adolescents* (TIEFBA; Fernández-Berrocal et al. 2011). The TIEFBA measures how well adolescents can use their EI skills to solve various emotional problems in real-life situations. The instrument was developed using the Situational Judgment Test approach, which involved five stages to ensure ecological validity (for more information on validity, see Fernández-Berrocal et al. 2011). Drawing from recent research on related measures (MacCann and Roberts 2008), TIEFBA utilizes a single situation that evokes emotions, providing the basis for various emotional tasks assessing the four branches of the EI model. There are eight emotionally eliciting scenes in the TIEFBA. Each scene contains two to three phrases that highlight the emotional component of an event involving one or more characters. Participants must complete four different scene activities to evaluate the four branches of the ability model of EI: Perceiving emotions, using emotions, understanding emotions, and managing emotions.

1. Perceiving emotions task: On a 5-point Likert scale (from 1 = “not at all” to 5 = “very much”), the adolescent is asked to evaluate the main protagonist’s facial expression. For example, how much anger, disgust, fear, happiness, sadness, and surprise does the character feel?
2. Using emotions task: On a 5-point Likert scale (from 1 = “not at all” to 5 = “very much”), the participant is asked to evaluate to what extent the main character’s mood would help them to perform three cognitive activities. This part assesses the participant’s understanding of how emotions are helpful in thinking and reasoning.
3. Understanding emotions task: On a 5-point Likert scale (from 1 = “not at all” to 5 = “very much”), the participant is asked to evaluate the extent to which four kinds of beliefs and thoughts are associated with the main character’s mood. This part rates the ability to link emotions with cognitive evaluations.
4. Managing emotions task: On a 5-point Likert scale (from 1 = “completely ineffective” to 5 = “completely effective”), the participant is asked to evaluate the efficacy of four alternative emotion-regulation strategies for reaching a specific goal. Based on four scenes, the participant must rate the effectiveness of the main characters’ emotional regulation strategies to achieve a goal (intrinsic regulation); then, in another four scenes, the participant must rate the efficacy of the strategies in which the main character regulates the emotion of other people to achieve a goal (extrinsic regulation).

The following seven scores are calculated by summing the participant’s performance on each activity across the eight scenes: A total score, which summarizes the participant’s performance across the four tasks; four scores corresponding to the four branches (Perceiving emotions, Using emotions, Understanding emotions, and Managing emotions); and two area scores corresponding to the experiential area (Perceiving and Using emotions tasks) and the strategic area (Understanding and Managing emotions task). However, we were only interested in the four branches for our study. The time taken to complete the test was 20–30 min. McDonald’s omega’s reliability coefficients in our sample were Perceiving = 0.83, Using = 0.80, Understanding = 0.73, Managing = 0.86, and Total Ability EI = 0.88. Contact *Fundación Botín* for further details on how to obtain the TIEFBA (http://www.fundacionbotin.org/educacion-contenidos/test-inteligencia-emocional.html, accessed on 8 August 2023). 

*Positive and Negative Affect Schedule* (PANAS; Watson et al. 1988). This is a self-report instrument composed of 20 items scored on a 5-point scale (1 “not at all” to 5 “strongly”). The questionnaire is designed to evaluate two emotional experience dimensions: PA, the intensity of positive mood states (e.g., interested, excited, proud), and NA, the intensity of negative mood states (e.g., upset, alert, scared). For our study, the Spanish version of the PANAS was used (Sandín et al. 1999) and the questionnaire asked participants “to what extent do you generally experience the following emotional states.” The McDonald’s omega reliability coefficient in our sample was 0.79 for PA and 0.82 for NA. 

*Subjective Happiness Scale* (SHS; Lyubomirsky and Lepper 1999). This 4-item Likert-scale measures global subjective happiness using statements with which participants either self-rate or compare themselves to others. The first item assesses the degree to which the respondent thinks they are happy (from 1 = not a very happy person to 7 = a very happy person). Item 2 assesses the person’s level of happiness in comparison to others (from 1 = less happy to 7 = happier). Item 3 assesses how frequently the person feels very happy, and Item 4 evaluates the opposite, that is, how frequently the person feels very unhappy (responses to both items ranging from 1 = not at all to 7= a great deal). McDonald’s omega in our sample was 0.72.

### 2.4. Data Analysis

First, descriptive statistics were used to examine each measure, and *t*-tests were used to analyze gender differences. Second, Pearson’s correlations were computed to examine the association between the Total Ability EI, PA, NA, and Subjective Happiness scores. Third, a path analysis was conducted to determine the direct and indirect relationships between Total Ability EI and Subjective Happiness via PA and NA. Total Ability EI was included as a predictor, PA and NA as intermediary variables, and Subjective Happiness as the criterion. Prior to the path analysis, we used regression analysis to examine whether gender acted as a moderator in each of the relationships included in the model. In the case of significance, the interaction was introduced into the path model. Predictors involved in the interaction were mean-centered. Fourth, a more complex model was constructed, including the four branches of TIEFBA as predictor variables (the rest of the path model was similar to the previous model). The indirect effects of the path analysis were examined using bias-corrected bootstrapping with 5000 iterations and 95% confidence intervals (CIs). Descriptive statistics, *t*-tests, and correlations were conducted using JAMOVI 2.3.21, while IBM SPSS AMOS 26.0 software (IBM Corp., Armonk, NY, USA) was utilized for path analyses. 

## 3. Results

Descriptive results and all Pearson’s correlations between all dimensions of the TIEFBA, Subjective Happiness, PA, and NA are presented in Table 1 and Table 2, respectively. According to the aims of this study, we can highlight a number of significant relationships. Total Ability EI correlated positively with Subjective Happiness; Total Ability EI correlated positively with PA and negatively with NA, while Subjective Happiness correlated positively with PA and negatively with NA (all *p* > .05). Finally, regarding the EI branches, Perceiving correlated negatively with NA, and Managing correlated positively with PA (all *p* > .05). Student’s *t*-test (Table 1) revealed that females obtained higher scores than males on Total Ability EI and the branches of Perceiving, Using, and Managing (all *p* < 0.05). However, no gender differences were found for the Understanding EI branch, PA, NA, and Subjective Happiness (*p* > .05).

Prior to conducting the path analyses, and given the gender differences observed in EI, we decided to examine the moderating effect of gender on the relationships of interest to include in the path models. These analyses revealed that gender moderated the relationship between Total Ability EI and Subjective Happiness (interaction effect: *b* = −0.03, *β* = −0.22, 95% *CI* [−0.049, −0.008]), which was significant for females (*b* = 0.04, *β* = 0.44, 95% *CI* [0.031, 0.063]) but not for males (95% *CI* [−0.001, 0.028]). With respect to EI branches, gender moderated the relationship between Using and Subjective Happiness (interaction effect: *b* = −0.02, *β* = −0.18, 95% *CI* [−0.039, −0.004]), which was significant for females (*b* = 0.02, *β* = 0.24, 95% *CI* [0.008, 0.030]), but not for males (95% *CI* [−0.013, 0.010]). The remaining relationships of interest (others included in the proposed path models) were not significantly moderated by gender.

**Figure 1:**
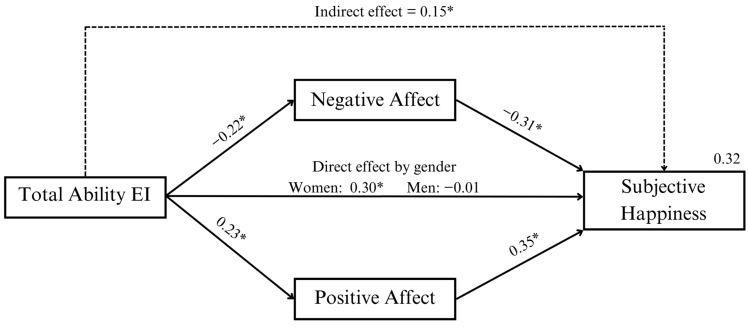
Graphical representation of the model analyzing EI as a total score, including standardized path coefficients and correlation coefficients. Note: An asterisk indicates significance at the *p* < 0.05 level.

Next, we conducted the analysis for the simple path model (see Figure 1). Following the previous analyses, the moderating effect of gender on the relationship between Total Ability EI and Subjective Happiness was introduced in the model, as this was previously found to be significant. The path analysis revealed a positive direct effect of Total Ability EI on Subjective Happiness (direct effect: *b* = 0.03, *β* = 0.31, 95% *CI* [0.013, 0.040]), but the Gender X Total Ability EI interaction for Subjective Happiness was also significant (direct effect: *b* = −0.03, *β* = −0.21, 95% *CI* [−0.042, −0.008]). A follow-up analysis of this interaction revealed that the positive direct effect of Total Ability EI on Subjective Happiness was only observed in females (direct effect: *b* = 0.03, *β* = 0.30, 95% *CI* [0.017, 0.045]), but not in males (95% *CI* [−0.015, 0.012]). Concerning the indirect effects, the analysis revealed a positive total indirect effect (via NA and PA jointly) of Total Ability EI on Subjective Happiness (indirect effect: *b* = 0.01, *β* = 0.15, 95% *CI* [0.010, 0.019]). Breaking down the total indirect effect into specific indirect effects revealed that Subjective Happiness was predicted by a positive specific indirect effect of Total Ability EI via NA (*b* = 0.01, *β* = 0.07, 95% *CI* [0.003, 0.010]) and PA (*b* = 0.01, *β* = 0.08, 95% *CI* [0.005, 0.012]). This model explained 32% of the variance in Subjective Happiness.

**Figure 2:**
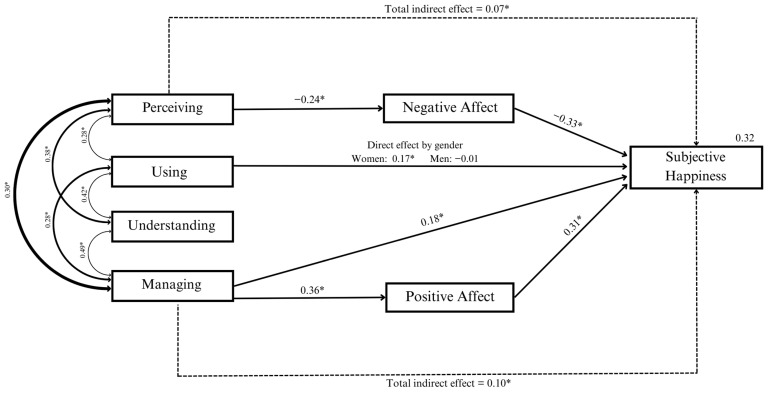
Graphical representation of the model analyzing the four EI branches, including standardized path coefficients and correlation coefficients. For easier interpretation of the results, only significant paths are shown. Note: An asterisk indicates significance at the *p* < 0.05 level.

Finally, we conducted the path model including EI branches (see Figure 2). The moderating effect of gender on the relationship between the Using branch and Subjective Happiness was included in the model, as this was significant. The analysis revealed a significant positive direct effect of Using and Managing on Subjective Happiness (Using: *b* = 0.01, *β* = 0.13, 95% *CI* [0.001, 0.017]; Managing: *b* = 0.01, *β* = 0.18, 95% *CI* [0.008, 0.023]) and a significant interaction between Gender and Using on Subjective Happiness (*b* = −0.02, *β* = −0.20, 95% *CI* [−0.036, −0.010]). Further analysis of this interaction revealed a positive direct effect of Using on Subjective Happiness in females (direct effect: *b* = 0.01, *β* = 0.17, 95% *CI* [0.004, 0.022]), but not in males (95% *CI* [−0.015, 0.002]). Regarding the indirect effects, the analysis revealed total positive indirect effects of Perceiving and Managing on Subjective Happiness via NA and PA jointly (Perceiving: *b* = 0.01, *β* = 0.07, 95% *CI* [0.002, 0.010]; Managing: *b* = 0.01, *β* = 0.10, 95% *CI* [0.04, 0.012]). For Perceiving, the analysis of specific indirect effects revealed a positive indirect effect via NA (*b* = 0.01, *β* = 0.08, 95% CI [0.004, 0.010]), but not via PA (95% *CI* [−0.004, 0.002]); while for Managing, a positive indirect effect was observed via PA (b = 0.01, *β* = 0.11, 95% *CI* [0.006, 0.013]), but not via NA (95% *CI* [−0.003, 0.002]). None of the remaining direct and indirect effects were significant. The model explained 32% of the variance in Subjective Happiness.

## 4. Discussion

Adolescence has increasingly been shown to be a risk period for suffering affective disorders and other mental health problems that impact the individual’s subjective well-being. This study aimed to analyze the relationship between performance-based ability emotional intelligence (ability EI) and Subjective Happiness through the mediating role of Positive (PA) and Negative Affect (NA) in adolescents. Furthermore, this association is explored through different dimensions of ability EI. This is the first study to measure this relationship in adolescents using a performance-ability EI instrument.

Concerning the descriptive results for gender, we found significant differences in EI scores but not in subjective well-being variables (PA, NA, and Subjective Happiness). In general, females scored higher on EI than males across all its dimensions (with Managing being the most notable) except for Understanding. These data are consistent with previous literature indicating that females score higher on EI measured with ability instruments (Fernández-Berrocal et al. 2018). Relating to subjective well-being, we found no significant gender differences. It appears that females begin to score lower on well-being from the age of 13 (Esteban-Gonzalo et al. 2020). Thus, our non-significant differences could be explained due to the age of our sample (12 years).

In line with our first hypothesis, we found a significant positive association between Total Ability EI and Subjective Happiness (Table 1), that is, adolescents that present higher EI perceive themselves as happier. These results are consistent with the findings of previous research (Abdollahi et al. 2015; Platsidou 2013; Tejada-Gallardo et al. 2022). In addition, it is known that happier people are less likely to have mental health problems or commit suicide (Domínguez-García and Fernández-Berrocal 2018; Fernández-Berrocal et al. 2006; Gómez-Baya et al. 2017). However, when we analyzed this relationship in the path model considering PA and NA, we found a direct effect of Total Ability EI on Subjective Happiness, which was only observed in females (Figure 1). While further research is still needed to establish clear relationships and draw firm conclusions, we can speculate about several factors that may contribute to the observed differences between females and males. Methodologically, females scored higher in Total Ability EI than males, which could potentially strengthen the association with Subjective Happiness. Another possible explanation is the influence of gender-based emotional socialization, where individuals are taught to approach their emotions differently based on their gender. Additionally, the distinct social demands and expectations placed on males and females may also be of relevance. For instance, females are often expected to display warmth, happiness, and emotional openness during social interactions (Keltner 1995; Smith et al. 2015). It has been observed that popular and well-liked girls often excel in verbal expression, possess an understanding of group dynamics, display lower levels of aggression, and show a keen interest in social relationships, particularly with boys (Brody 2000). It is likely that utilizing EI as a tool to navigate these social challenges directly influences their perception of happiness. To further investigate this relationship, future studies should continue using performance-based EI instruments and examining it across different age groups.

Concerning our second hypothesis, we found a significant relationship between Total Ability EI and affect components; specifically, this relationship was positive for PA and negative for NA. This indicates that emotionally intelligent adolescents have greater PA and less NA, as confirmed by various investigations (Gómez-Baya and Mendoza 2018; MacCann et al. 2020; Zhao et al. 2020). Since we used the PANAS instrument, this specifically means that students who scored lower on ability EI experienced a lower intensity of positive emotions and a greater intensity of negative emotions.

Regarding our third hypothesis, it was found that PA and NA were positively and negatively associated with happiness, respectively, so participants who experienced more PA and less NA perceived themselves as happier. These findings coincide with those of previous studies (Bhutoria and Hooja 2018; Cheng and Furnham 2003; Lyubomirsky et al. 2005; Singh and Jha 2008) and highlight how the intensity of the type of emotions experienced (pleasant or unpleasant) can impact the subjective happiness experienced by the individual. 

Confirming hypothesis four, Total Ability EI had a positive indirect effect on Subjective Happiness via PA and NA jointly (Figure 1). Specifically, the most emotionally intelligent adolescents tend to perceive more intense positive and less intense negative emotions, which seems to be related to greater subjective happiness. This result could be taken to indicate a protective role of EI in adolescents’ subjective well-being by regulating the intensity of their pleasant or unpleasant emotions. This finding is consistent with previous research indicating that affectivity plays a significant role in shaping evaluations of life satisfaction (Extremera and Rey 2016; Kuppens et al. 2008, Schwarz and Clore 2007). According to the affect-as-information perspective, individuals commonly utilize their affective balance as a source of information when assessing their overall life satisfaction (Schwarz and Clore 2007). In summary, individuals with high EI may experience better subjective well-being by effectively utilizing their emotional strategies to modify the intensity of both pleasant and unpleasant emotions. 

Related to hypothesis four, a more detailed analysis of TIEFBA branches (Figure 2), revealed that Perceiving, one of the earliest and most basic emotional abilities, showed a significant negative indirect effect on ability EI through NA but not PA. The greater the ability to perceive emotions, the lower the intensity of negative emotions, which positively impacts Subjective Happiness. This implies that sensitivity to emotional cues is important for negative but not positive affect. Perceiving our environment appropriately could prevent us from misinterpreting contextual situations, allowing us to identify and address them promptly before negative emotions increase (MacCann et al. 2020). Regarding Managing, we observed both direct and indirect effects on Subjective Happiness through PA (Quoidbach et al. 2010). EI comprises different dimensions that increase in difficulty and are interdependent. Managing emotions is the strategy that requires the most resources to be efficient and usually has the greatest impact on health variables (Hu et al. 2014), and at the same time, this is the strategy that is most susceptible to improvement by the individual. Adolescents who perceive themselves as happier tend to regulate their emotions to experience more intense positive emotions. In this sense, it is logical to suppose that even when an adolescent effectively manages their emotions and experiences happiness, they may still feel negative emotions to a certain extent. While adolescents sometimes have no control over the negative events that happen to them or around them, they may enhance their well-being by actively creating positive experiences and balancing these with negative experiences. These findings are consistent with those reported by MacCann et al. (2020), who concluded that all EI abilities may contribute to reducing the impact of negative affect (down-regulation), while management specifically contributes to enhancing positive affect (up-regulation). Understanding this direct and indirect relationship is important because it provides insights into which EI abilities should be targeted for improving the subjective well-being of adolescents. Finally, gender moderated the relationship between Using and Subjective Happiness, which was significant for females but not males. In this study, female adolescents showed three direct effects regarding the relationship with Subjective Happiness (Total Ability EI, Using, and Managing). It appears that for females, the influence of ability EI on their Subjective Happiness is stronger and does not necessarily depend on the modulation of their affect. Further research is needed to deepen our understanding of how gender in adolescents interacts with various branches of ability EI in relation to well-being.

While this study helps to understand the mechanisms underlying adolescents’ subjective well-being through performance EI measures, it is not exempt from limitations. The average age of our sample is 12 years, and studies on the behavior of these variables should be conducted with a range of ages throughout adolescence. Moreover, because the nature of the study is cross-sectional and correlational, causal mechanisms cannot be established. Future research should use experimental and longitudinal methods to examine the causal relationship between our target variables and adolescents’ happiness. In addition, longitudinal and experimental studies should examine the effects of EI training on adolescent well-being to inform the development of improved programs and to apply our findings to a more real-world context (Morrish et al. 2018). More research is also needed to provide consistent data on how various EI abilities could differ according to gender or other variables such as ethnicity or socioeconomic and socio-educational level (Gutiérrez-Cobo et al. 2023b). Finally, it would be useful to conduct similar studies using different samples, such as those recruited from clinical populations.

In conclusion, it appears that EI—through PA and NA—can play an important role in how happy adolescents perceive themselves. Given the most recent data on suicide, depression, and other affective disorders, our findings emphasize the potential of using EI training as an intervention and prevention tool for adolescent well-being, which is currently an essential global priority. While the adolescent environment cannot be controlled to manipulate how many positive or negative emotions they experience daily, there is a tool at our disposal that can mitigate or improve the intensity of these feelings to positively affect their happiness. However, when designing an intervention, it is crucial to understand the specific needs of the targeted individuals and determine how to address them effectively. In this regard, this study offers valuable insights for developing programs aimed at adolescents. For instance, training in perception ability could lead to a reduction in negative affect, making it beneficial for interventions aimed at alleviating stress and social anxiety. On the other hand, emotion management training can help to increase the intensity of positive emotions, thus contributing to interventions focused on positive well-being (MacCann et al. 2020). Therefore, it is important to emphasize the inclusion of performance-based instruments, in addition to self-report measures, in interventions. This approach will help to accurately identify the specific abilities that require attention in adolescents. Additionally, it is crucial to consider gender differences when designing and implementing such interventions.

## Figures and Tables

**Table 1:** Means, standard deviations (SD), minimum, maximum, and *t*-test for gender differences.

	Gender	Mean	SD	Minimum	Maximum	*t*-Test	Cohen’s d
Perceiving	M	98.68	14.85	48.05	121.82	−2.24 *	−0.25
	F	102.25	14.17	55.51	129.00		
Using	M	94.37	14.70	53.33	132.82	−2.02 *	−0.22
	F	97.73	15.66	59.45	132.47		
Understanding	M	96.13	13.45	65.15	140.43	−1.32	−0.15
	F	98.19	13.71	62.06	132.95		
Managing	M	97.12	13.64	65.15	128.65	−2.53 **	−0.28
	F	101.05	14.75	62.06	126.98		
Total Ability EI	M	95.71	13.41	58.23	135.21	−2.90 **	−0.32
	F	99.95	13.23	59.90	131.91		
Positive Affect	M	3.41	0.63	1.80	5.00	−0.68	−0.07
	F	3.46	0.64	1.80	4.80		
Negative Affect	M	1.97	0.65	1.00	5.00	−1.76	−0.19
	F	2.09	0.65	1.00	4.70		
Subjective Happiness	M	5.19	1.16	1.00	7.00	−0.44	−0.05
	F	5.25	1.27	1.00	7.00		

Note. M = Male (N = 171), F = Female (N = 162), SD = Standard Deviations. * *p* < .05, ** *p* < .001.

**Table 2:** Pearson’s correlations among the study variables.

	1	2	3	4	5	6	7	8
1. Perceiving	—							
2. Using	0.28 **	—						
3. Understanding	0.38 **	0.42 **	—					
4. Managing	0.30 **	0.28 **	0.49 **	—				
5. Total Ability EI	0.72 **	0.68 **	0.77 **	0.71 **	—			
6. Positive Affect	0.08 *	0.03 *	0.22 **	0.37 **	0.23 **	—		
7. Negative Affect	−0.26 **	−0.14 *	−0.13 *	−0.08 *	−0.22 **	−0.05 *	—	
8. Subjective Happiness	0.18 **	0.12 *	0.25 **	0.33 **	0.30 **	0.40 **	−0.37 **	—

Note. * *p* < .05, ** *p* < .001.

## Data Availability

Study data are available upon request from correspondence author Rosario Cabello (rcabello@uma.es).

## References

[B1-jintelligence-11-00166] Abdollahi Abbas, Talib Mansor Abu, Motalebi Seyedeh Ameneh (2015). Emotional Intelligence and Depressive Symptoms as Predictors of Happiness Among Adolescents. Iranian Journal of Psychiatry and Behavioral Sciences.

[B2-jintelligence-11-00166] Bar-On Reuven (1997). BarOn Emotional Quotient Inventory (BarOn EQ-i).

[B3-jintelligence-11-00166] Bhutoria Kanica, Hooja Himangini (2018). Role of positive affect and negative affect in orientation to happiness: A study on working population. Indian Journal of Health and Well-Being.

[B4-jintelligence-11-00166] Brackett Marc A., Rivers Susan E., Shiffman Sara, Lerner Nicole, Salovey Peter (2006). Relating emotional abilities to social functioning: A comparison of self-report and performance measures of emotional intelligence. Journal of Personality and Social Psychology.

[B5-jintelligence-11-00166] Brody Leslie R., Fischer Agneta H. (2000). The socialization of gender differences in emotional expression: Display rules, infant temperament, and differentiation. Gender and Emotion: Social Psychological Perspectives.

[B6-jintelligence-11-00166] Busseri Michael A., Sadava Stan W. (2011). A Review of the Tripartite Structure of Subjective Well-Being: Implications for Conceptualization, Operationalization, Analysis, and Synthesis. Personality and Social Psychology Review.

[B7-jintelligence-11-00166] Cabello Rosario, Fernandez-Berrocal Pablo (2015). Under which conditions can introverts achieve happiness? Mediation and moderation effects of the quality of social relationships and emotion regulation ability on happiness. PeerJ.

[B8-jintelligence-11-00166] Cabello Rosario, Sorrel Miguel A., Fernández-Pinto Irene, Extremera Natalio, Fernández-Berrocal Pablo (2016). Age and gender differences in ability emotional intelligence in adults: A cross-sectional study. Developmental Psychology.

[B9-jintelligence-11-00166] Casas Ferran, Figuer Cristina, González Mònica, Malo Sara, Alsinet Carles, Subarroca Sandra (2007). The Well-Being of 12- to 16-Year-Old Adolescents and their Parents: Results from 1999 to 2003 Spanish Samples. Social Indicators Research.

[B10-jintelligence-11-00166] Castillo-Gualda Ruth, Cabello Rosario, Herrero Marta, Rodríguez-Carvajal Raquel, Fernández-Berrocal Pablo (2018). A Three-Year Emotional Intelligence Intervention to Reduce Adolescent Aggression: The Mediating Role of Unpleasant Affectivity. Journal of Research on Adolescence.

[B11-jintelligence-11-00166] Cheng Helen, Furnham Adrian (2003). Personality, self-esteem, and demographic predictions of happiness and depression. Personality and Individual Differences.

[B12-jintelligence-11-00166] Chui Wing Hong, Wong Mathew Y. H. (2016). Gender Differences in Happiness and Life Satisfaction Among Adolescents in Hong Kong: Relationships and Self-Concept. Social Indicators Research.

[B13-jintelligence-11-00166] Crawford John R., Henry Julie D. (2004). The Positive and Negative Affect Schedule (PANAS): Construct validity, measurement properties and normative data in a large non-clinical sample. British Journal of Clinical Psychology.

[B14-jintelligence-11-00166] Daros Alexander R., Haefner Sasha A., Asadi Shayan, Kazi Sharifa, Rodak Terri, Quilty Lena C. (2021). A meta-analysis of emotional regulation outcomes in psychological interventions for youth with depression and anxiety. Nature Human Behaviour.

[B15-jintelligence-11-00166] Diener Ed, Suh Eunkook M., Lucas Richard E., Smith Heidi L. (1999). Subjective well-being: Three decades of progress. Psychological Bulletin.

[B16-jintelligence-11-00166] Diener Ed, Oishi Shigehiro, Tay Louis (2018). Advances in subjective well-being research. Nature Human Behaviour.

[B17-jintelligence-11-00166] Domínguez-García Elena, Fernández-Berrocal Pablo (2018). The Association Between Emotional Intelligence and Suicidal Behavior: A Systematic Review. Frontiers in Psychology.

[B18-jintelligence-11-00166] Durlak Joseph A., Mahoney Joseph L., Boyle Alaina E. (2022). What we know, and what we need to find out about universal, school-based social and emotional learning programs for children and adolescents: A review of meta-analyses and directions for future research. Psychological Bulletin.

[B19-jintelligence-11-00166] Esteban-Gonzalo Sara, Esteban-Gonzalo Laura, Cabanas-Sánchez Verónica, Miret Marta, Veiga Oscar L. (2020). The Investigation of Gender Differences in Subjective Wellbeing in Children and Adolescents: The UP&DOWN Study. International Journal of Environmental Research and Public Health.

[B20-jintelligence-11-00166] Extremera Natalio, Rey Lourdes (2016). Ability emotional intelligence and life satisfaction: Positive and negative affect as mediators. Personality and Individual Differences.

[B21-jintelligence-11-00166] Extremera Natalio, Fernández-Berrocal Pablo, Salovey Peter (2006). Spanish version of the Mayer-Salovey-Caruso Emotional Intelligence Test (MSCEIT). Version 2.0: Reliabilities, age and gender differences. Psicothema.

[B22-jintelligence-11-00166] Fernández-Berrocal Pablo (2015). Nuevos Instrumentos de Evaluación de la Inteligencia Emocional en la Infancia y la Adolescencia. Educación Emocional y Social: Análisis Internacional.

[B24-jintelligence-11-00166] Fernández-Berrocal Pablo, Ruiz-Aranda Desireé, Salguero José M., Palomera Raquel, Extremera Natalio (2018). The Relationship of Botín Foundation’s Emotional Intelligence Test (TIEFBA) with Personal and Scholar Adjustment of Spanish Adolescents. Revista de Psicodidáctica (English Ed.).

[B23-jintelligence-11-00166] Fernández-Berrocal Pablo, Extremera Natalio, Palomera Raquel, Ruiz-Aranda Desireé, Salguero José Martín (2011). Test de Inteligencia Emocional de la Fundación Botín para adolescentes (TIEFBA).

[B25-jintelligence-11-00166] Fernández-Berrocal Pablo, Alcaide Rocio, Extremera Natalio, Pizarro David (2006). The role of emotional intelligence in anxiety and depression among adolescents. Individual Differences Research.

[B26-jintelligence-11-00166] Goldbeck Lutz, Schmitz Tim G., Besier Tanja, Herschbach Peter, Henrich Gerhard (2007). Life satisfaction decreases during adolescence. Quality of Life Research.

[B28-jintelligence-11-00166] Gómez-Baya Diego, Mendoza Ramón (2018). Trait Emotional Intelligence as a Predictor of Adaptive Responses to Positive and Negative Affect During Adolescence. Frontiers in Psychology.

[B29-jintelligence-11-00166] Gómez-Baya Diego, Mendoza Ramon, Paino Susana, de Matos Margarida Gaspar (2017). Perceived emotional intelligence as a predictor of depressive symptoms during mid-adolescence: A two-year longitudinal study on gender differences. Personality and Individual Differences.

[B30-jintelligence-11-00166] Gómez-Leal Raquel, Gutiérrez-Cobo María J., Cabello Rosario, Megías Alberto, Fernández-Berrocal Pablo (2018). The Relationship Between the Three Models of Emotional Intelligence and Psychopathy: A Systematic Review. Frontiers in Psychiatry.

[B27-jintelligence-11-00166] Gong Zhun, Jiao Xinian (2019). Are Effect Sizes in Emotional Intelligence Field Declining? A Meta-Meta Analysis. Frontiers in Psychology.

[B31-jintelligence-11-00166] Gutiérrez-Cobo María J., Megías Alberto, Gómez-Leal Raquel, Cabello Rosario, Fernández-Berrocal Pablo (2018). The role of emotional intelligence and negative affect as protective and risk factors of aggressive behavior: A moderated mediation model. Aggressive Behavior.

[B32-jintelligence-11-00166] Gutiérrez-Cobo María J., Cabello Rosario, Fernández-Berrocal Pablo (2017). The Three Models of Emotional Intelligence and Performance in a Hot and Cool go/no-go Task in Undergraduate Students. Frontiers in Behavioral Neuroscience.

[B33-jintelligence-11-00166] Gutiérrez-Cobo María José, Megías-Robles Alberto, Gómez-Leal Raquel, Cabello Rosario, Fernández-Berrocal Pablo (2021). Is It Possible to Be Happy during the COVID-19 Lockdown? A Longitudinal Study of the Role of Emotional Regulation Strategies and Pleasant Activities in Happiness. International Journal of Environmental Research and Public Health.

[B34-jintelligence-11-00166] Gutiérrez-Cobo María José, Megías-Robles Alberto, Gómez-Leal Raquel, Cabello Rosario, Fernández-Berrocal Pablo (2023a). Emotion regulation strategies and aggression in youngsters: The mediating role of negative affect. Heliyon.

[B35-jintelligence-11-00166] Gutiérrez-Cobo María José, Cabello Rosario, Megías-Robles Alberto, Gómez-Leal Raquel, Baron-Cohen Simon, Fernández-Berrocal Pablo (2023b). Does our cognitive empathy diminish with age? The moderator role of educational level. International Psychogeriatrics.

[B36-jintelligence-11-00166] Hu Tianqiang, Zhang Dajun, Wang Jinliang, Mistry Ritesh, Ran Guangming, Wang Xinqiang (2014). Relation between Emotion Regulation and Mental Health: A Meta-Analysis Review. Psychological Reports.

[B37-jintelligence-11-00166] Inglés Cándido J., Díez María Soledad Torregrosa, Fernández José Manuel García, Monteagudo Mari Carmen Martínez, López Estefanía Estévez, Domenech Beatriz Delgado (2014). Conducta agresiva e inteligencia emocional en la adolescencia. European Journal of Educational Psychology.

[B38-jintelligence-11-00166] Joseph Dana L., Newman Daniel A. (2010). Emotional intelligence: An integrative meta-analysis and cascading model. Journal of Applied Psychology.

[B39-jintelligence-11-00166] Keltner Dacher (1995). Signs of appeasement: Evidence for the distinct displays of embarrassment, amusement, and shame. Journal of Personality and Social Psychology.

[B40-jintelligence-11-00166] Kong Feng, Zhao Jingjing (2013). Affective mediators of the relationship between trait emotional intelligence and life satisfaction in young adults. Personality and Individual Differences.

[B41-jintelligence-11-00166] Koydemir Selda, Schütz Astrid (2012). Emotional intelligence predicts components of subjective well-being beyond personality: A two-country study using self- and informant reports. The Journal of Positive Psychology.

[B42-jintelligence-11-00166] Kuppens Peter, Realo Anu, Diener Ed (2008). The role of positive and negative emotions in life satisfaction judgment across nations. Journal of Personality and Social Psychology.

[B43-jintelligence-11-00166] Llamas-Díaz Desirée, Cabello Rosario, Megías-Robles Alberto, Fernández-Berrocal Pablo (2022). Systematic review and meta-analysis: The association between emotional intelligence and subjective well-being in adolescents. Journal of Adolescence.

[B44-jintelligence-11-00166] Lyubomirsky Sonja, Lepper Heidi S. (1999). A Measure of Subjective Happiness: Preliminary Reliability and Construct Validation. Social Indicators Research.

[B45-jintelligence-11-00166] Lyubomirsky Sonja, King Laura, Diener Ed (2005). The Benefits of Frequent Positive Affect: Does Happiness Lead to Success?. Psychological Bulletin.

[B46-jintelligence-11-00166] MacCann Carolyn, Roberts Richard D. (2008). New paradigms for assessing emotional intelligence: Theory and data. Emotion.

[B47-jintelligence-11-00166] MacCann Carolyn, Erbas Yasemin, Dejonckheere Egon, Minbashian Amirali, Kuppens Peter, Fayn Kirill (2020). Emotional intelligence relates to emotions, emotion dynamics, and emotion complexity: A meta-analysis and experience sampling study. European Journal of Psychological Assessment.

[B48-jintelligence-11-00166] Martínez-Monteagudo María Carmen, García-Sancho Esperanza, Delgado Beatriz (2019). The mediating role of affect in the relationship between emotional intelligence and academic performance in university students. Social Psychology of Education.

[B49-jintelligence-11-00166] Mayer John D., Salovey Peter, Sluyter David (1997). What is emotional intelligence?. Emotional Development and Emotional Intelligence: Educational Implications.

[B50-jintelligence-11-00166] Mayer John D., Salovey Peter, Caruso David R. (2002). MSCEIT: Mayer-Salovey-Caruso Emotional Intelligence Test.

[B53-jintelligence-11-00166] Mayer John D., Salovey Peter, Caruso David R. (2008). Emotional intelligence: New ability or eclectic traits?. American Psychologist.

[B51-jintelligence-11-00166] Mayer John D., Salovey Peter, Caruso David R., Cherkasskiy Lillia (1997). What is emotional intelligence?. Emotional Development and Emotional Intelligence: Educational Implications.

[B52-jintelligence-11-00166] Mayer John D., Salovey Peter, Caruso David R., Sternberg Robert J. (2000). Models of emotional intelligence. Emotional Intelligence: Key Readings on the Mayer and Salovey Model.

[B54-jintelligence-11-00166] Megías Alberto, Gómez-Leal Raquel, Gutiérrez-Cobo María José, Cabello Rosario, Fernández-Berrocal Pablo (2018). The relationship between aggression and ability emotional intelligence: The role of negative affect. Psychiatry Research.

[B55-jintelligence-11-00166] Moksnes Unni K., Espnes Geir A. (2013). Self-esteem and life satisfaction in adolescents—Gender and age as potential moderators. Quality of Life Research.

[B56-jintelligence-11-00166] Morrish Lucy, Rickard Nikki, Chin Tan Chyuan, Vella-Brodrick Dianne Anne (2018). Emotion Regulation in Adolescent Well-Being and Positive Education. Journal of Happiness Studies.

[B57-jintelligence-11-00166] O’Boyle Ernest H., Humphrey Ronald H., Pollack Jeffrey M., Hawver Thomas H., Story Paul A. (2011). The relation between emotional intelligence and job performance: A meta-analysis. Journal of Organizational Behavior.

[B58-jintelligence-11-00166] Palmer Benjamin R., Gignac Gilles, Manocha Ramesh, Stough Con (2005). A psychometric evaluation of the Mayer–Salovey–Caruso Emotional Intelligence Test Version 2.0. Intelligence.

[B59-jintelligence-11-00166] Petrides Konstantinos V., Furnham Adrian (2000). Gender Differences in Measured and Self-Estimated Trait Emotional Intelligence. Sex Roles.

[B60-jintelligence-11-00166] Petrides Konstantinos V., Sangareau Yolanda, Furnham Adrian, Frederickson Norah (2006). Trait Emotional Intelligence and Children’s Peer Relations at School. Social Development.

[B61-jintelligence-11-00166] Platsidou Maria (2013). Trait emotional intelligence predicts happiness, but how? An empirical study in adolescents and young adults. International Journal of Well-Being.

[B62-jintelligence-11-00166] Quoidbach Jordi, Berry Elizabeth V., Hansenne Michel, Mikolajczak Moïra (2010). Positive emotion regulation and well-being: Comparing the impact of eight savoring and dampening strategies. Personality and Individual Differences.

[B63-jintelligence-11-00166] Resurrección Davinia María, Salguero José Martín, Ruiz-Aranda Desireé (2014). Emotional intelligence and psychological maladjustment in adolescence: A systematic review. Journal of Adolescence.

[B64-jintelligence-11-00166] Salavera Carlos, Usán Pablo (2020). The Mediating Role of Affects between Mind-Wandering and Happiness. Sustainability.

[B65-jintelligence-11-00166] Salguero José M., Extremera Natalio, Fernández-Berrocal Pablo (2012). Emotional intelligence and depression: The moderator role of gender. Personality and Individual Differences.

[B66-jintelligence-11-00166] Salguero José M., Fernández-Berrocal Pablo, Balluerka Nekane, Aritzeta Aitor (2010). Measuring Perceived Emotional Intelligence in the Adolescent Population: Psychometric Properties of the Trait Meta-Mood Scale. Social Behavior and Personality: An International Journal.

[B67-jintelligence-11-00166] Salovey Peter, Mayer John D. (1990). Emotional Intelligence. Imagination, Cognition and Personality.

[B68-jintelligence-11-00166] Salovey Peter, Mayer John D., Goldman Susan Lee, Turvey Carolyn, Palfai Tibor P., Pennebaker James W. (1995). Emotional attention, clarity, and repair: Exploring emotional intelligence using the Trait Meta-Mood Scale. Emotion, Disclosure, & Health.

[B71-jintelligence-11-00166] Sánchez-Álvarez Nicolás, Extremera Natalio, Fernández-Berrocal Pablo (2015). Maintaining Life Satisfaction in Adolescence: Affective Mediators of the Influence of Perceived Emotional Intelligence on Overall Life Satisfaction Judgments in a Two-Year Longitudinal Study. Frontiers in Psychology.

[B72-jintelligence-11-00166] Sánchez-Álvarez Nicolás, Extremera Natalio, Fernández-Berrocal Pablo (2016). The relation between emotional intelligence and subjective well-being: A meta-analytic investigation. The Journal of Positive Psychology.

[B69-jintelligence-11-00166] Sanchez-Ruiz Maria-Jose, Baaklini Amal (2018). Individual and social correlates of aggressive behavior in Lebanese undergraduates: The role of trait emotional intelligence. The Journal of Social Psychology.

[B70-jintelligence-11-00166] Sandín Bonifacio, Chorot Paloma, Lostao Lourdes, Joiner Thomas E., Santed Miguel A., Valiente Rosa M. (1999). Escalas PANAS de afecto positivo y negativo: Validación factorial y convergenciatranscultural. Psicothema.

[B73-jintelligence-11-00166] Schimmack Ulrich, Oishi Shigehiro, Furr R. Michael, Funder David C. (2004). Personality and Life Satisfaction: A Facet-Level Analysis. Personality and Social Psychology Bulletin.

[B74-jintelligence-11-00166] Schwarz Norbert, Clore Gerald L., Kruglanski Arie W., Higgins E. Tory (2007). Feelings and phenomenal experiences. Social Psychology: Handbook of Basic Principles.

[B75-jintelligence-11-00166] Siegling Alexander B., Saklofske Donald H., Petrides Konstantinos V., Boyle Gregory J., Saklofske Donald H., Matthews Gerald (2015). Measures of Ability and Trait Emotional Intelligence. Measures of Personality and Social Psychological Constructs.

[B76-jintelligence-11-00166] Singh Kamlesh, Jha Shalini Duggal (2008). Positive and negative affect and grit as predictors of happiness and life satisfaction. Journal of the Indian Academy of Applied Psychology.

[B77-jintelligence-11-00166] Smith Jacqueline S., LaFrance Marianne, Knol Kevin H., Tellinghuisen Donald J., Moes Paul (2015). Surprising Smiles and Unanticipated Frowns: How Emotion and Status Influence Gender Categorization. Journal of Nonverbal Behavior.

[B78-jintelligence-11-00166] Taylor Rebecca D., Oberle Eva, Durlak Joseph A., Weissberg Roger P. (2017). Promoting Positive Youth Development Through School-Based Social and Emotional Learning Interventions: A Meta-Analysis of Follow-Up Effects. Child Development.

[B79-jintelligence-11-00166] Tejada-Gallardo Claudia, Blasco-Belled Ana, Torrelles-Nadal Cristina, Alsinet Carles (2022). How does emotional intelligence predict happiness, optimism, and pessimism in adolescence? Investigating the relationship from the bifactor model. Current Psychology.

[B80-jintelligence-11-00166] UNICEF (2021). Estado Mundial de la Infancia 2021. En mi Mente.

[B81-jintelligence-11-00166] UNICEF (2022). 10 de Octubre. Día Mundial de la Salud Mental.

[B82-jintelligence-11-00166] Vega Alberto, Cabello Rosario, Megías-Robles Alberto, Gómez-Leal Raquel, Fernández-Berrocal Pablo (2022). Emotional Intelligence and Aggressive Behaviors in Adolescents: A Systematic Review and Meta-Analysis. Trauma, Violence, & Abuse.

[B83-jintelligence-11-00166] Watson David, Clark Lee Anna, Tellegen Auke (1988). Development and validation of brief measures of positive and negative affect: The PANAS scales. Journal of Personality and Social Psychology.

[B84-jintelligence-11-00166] Webb Christian A., Schwab Zachary J., Weber Mareen, DelDonno Sophie, Kipman Maia, Weiner Melissa R., Killgore William D.S. (2013). Convergent and divergent validity of integrative versus mixed model measures of emotional intelligence. Intelligence.

[B85-jintelligence-11-00166] Williams John (2008). The Declaration of Helsinki and public health. Bulletin of the World Health Organization.

[B86-jintelligence-11-00166] Zeidner Moshe, Matthews Gerald, Shemesh Dorit Olenik (2016). Cognitive-Social Sources of Wellbeing: Differentiating the Roles of Coping Style, Social Support and Emotional Intelligence. Journal of Happiness Studies.

[B87-jintelligence-11-00166] Zeidner Moshe, Matthews Gerald, Roberts Richard D. (2012). What We Know about Emotional Intelligence: How It Affects Learning, Work, Relationships, and Our Mental Health.

[B88-jintelligence-11-00166] Zhao Jia-Lin, Cai Dan, Yang Cai-Yun, Shields John, Xu Zhe-Ning, Wang Chun-Ying (2020). Trait emotional intelligence and young adolescents’ positive and negative affect: The mediating roles of personal resilience, social support, and prosocial behavior. Child & Youth Care Forum.

